# Mexican Spanish adaptation for the Affective Norms for English Words (ANEW)

**DOI:** 10.3758/s13428-025-02703-5

**Published:** 2025-05-12

**Authors:** Vladimir Huerta-Chavez, Luis A. Llamas-Alonso, Armando Quetzalcóatl Angulo-Chavira

**Affiliations:** 1 Instituto de Neurociencias, Universidad de Guadalajara, Guadalajara, México; 2 Facultad de Ciencias Administrativas y Sociales, Universidad Autónoma de Baja California, Ensenada, México; 3 Facultad de Psicología, Universidad Nacional Autónoma de México, Mexico, México

**Keywords:** Affective norms, Mexican-Spanish, Emotional word ratings, Valence-arousal-dominance, Psycholinguistics

## Abstract

This study adapts the Affective Norms for English Words (ANEW) dataset for Mexican Spanish, validating emotional dimensions in culturally relevant contexts. A total of 753 participants rated 1,028 translated words on valence, arousal, and dominance using the Self-Assessment Manikin (SAM) scale. The adaptation ensured linguistic equivalence through iterative translation and consensus processes, selecting region-specific terms verified with the Corpus XXI of the Royal Spanish Academy. Split-half correlations confirmed high internal consistency across dimensions, demonstrating stable and reliable ratings within the Mexican sample. Cross-linguistic analyses revealed strong correlations between Mexican Spanish and norms for European Portuguese and Spanish, with moderate correlations to English norms, highlighting cultural and linguistic influences on emotional word ratings. Gender differences further provided insights into demographic factors affecting emotional word processing. These findings underscore the need for culturally specific adaptations in research, ensuring that affective norms align with regional language use and emotional perception. This study offers a methodological framework applicable to other linguistic and cultural contexts, enhancing the precision of cross-cultural research in affective science.

## Introduction

Emotions are fundamental to daily life, playing a vital role in expressing our feelings and understanding those of others. These emotions are conveyed through various modalities, including body language, facial expressions, prosody, verbal communication, and written language. Among these, the ability to distinguish emotions in language is particularly critical for successful social adaptation (Israelashvili & Fischer, 2023). Written language, in particular, provides a unique lens for studying emotional processing in isolation, as it eliminates the influence of nonverbal cues like body language and tone of voice. Moreover, it offers precise control over key linguistic variables, such as age of language acquisition, word frequency (Monnier & Syssau, 2014), and word length, enhancing the replicability of research findings. This precise control is increasingly significant in the context of digital communication, where written language has become a dominant medium for expressing emotions.

Emotional words can be broadly divided into two categories: those that explicitly label emotions (e.g., “happiness” or “fear”) and those that carry emotional connotations (e.g., “murder” or “chocolate”) through association (Wu & Zhang, 2020). To systematically analyze these words, affective word norms, such as the Affective Norms for English Words (ANEW) dataset (Bradley & Lang, 1999), classify them along three key affective dimensions: valence, arousal, and dominance. Valence indicates whether a stimulus is perceived as positive or negative (Barrett, 2006), arousal reflects the intensity of the emotional response, and dominance captures the sense of control or power individuals experience over the emotion elicited by the word (Jerram et al., 2014). This classification provides a structured framework for examining the emotional impact of language. The quantitative assessment of these affective categories has been made possible through the Self-Assessment Manikin (SAM) scale, a pictorial, nonverbal evaluation technique that measures an individual's affective response to a wide range of stimuli across various contexts. This tool is based on a dimensional approach encompassing the dimensions of valence, arousal, and dominance (Bradley & Lang, 1994). These affective dimensions are shaped by cultural factors, including societal norms and regional linguistic nuances (Grossmann et al., 2012; Kuppens et al., 2017; Russell, 1991).

Consequently, adapting affective word norms to diverse languages and cultural contexts is crucial. Numerous ANEW adaptations have emerged, including British English and Finnish (Eilola & Havelka, 2010), French (Monnier & Syssau, 2014), German (Schmidtke et al., 2014), Turkish (Torkamani-Azar et al., 2019), Polish (Imbir, 2015), European Portuguese (Soares et al., 2012), Brazilian Portuguese (Kristensen et al., 2011), Italian (Montefinese et al., 2014), European Spanish (Redondo et al., 2007), and Argentine Rioplatense Spanish (Sarli & Justel, 2022).

Typically, these studies translate words, collect affective ratings from native speakers, and run statistical analyses to verify the reliability of emotional dimensions. Although cross-language valence ratings are consistently high, studies often reveal greater cultural variability for arousal and dominance. For instance, while Eilola and Havelka (2010) found similar valence patterns between American and British English, Finnish speakers judged words deemed highly intense in English as less arousing. Monnier and Syssau (2014) similarly reported robust valence convergence for French but noted reduced perceived emotional intensity (arousal). Likewise, Italian (Montefinese et al., 2014), German (Schmidtke et al., 2014), Turkish (Torkamani-Azar et al., 2019), and Polish (Imbir, 2015) studies all showed strong valence alignment with American English norms yet revealed distinct cultural differences in arousal and sometimes dominance, particularly for negative words, which tended to be rated as more arousing or more culturally specific in each language.

Even within the same language, cross-cultural variations emerge in ANEW adaptations, as illustrated by the Portuguese and Spanish cases. For Portuguese, Kristensen et al. (2011) and Soares et al. (2012) adapted ANEW to Brazilian and European variants, respectively, both confirming a strong valence correlation with American norms and reproducing the classic “boomerang-shaped” valence–arousal pattern. Yet in both adaptations, words were perceived with lower arousal than in English, suggesting a weaker sense of emotional intensity. This effect was more pronounced in European Portuguese, where lower dominance ratings further indicated that participants felt less in control than their American counterparts. Meanwhile, Brazilian Portuguese ratings tended to align more closely with American English. Similarly, Spanish adaptations by Redondo et al. (2007) for European Spanish and Sarli and Justel (2022) for Argentine Rioplatense Spanish found high valence correlations and the typical “boomerang-shaped” distribution, but revealed notable differences. Argentinian participants rated negative and neutral words as more arousing and reported a stronger feeling of control for both positive and negative words. In contrast, European Spanish speakers gave lower arousal scores and perceived less control for negative words. Additionally, no gender differences were reported in European Spanish, whereas in Argentina, women rated negative words as more arousing and positive words as more pleasant, and felt more in control of positive words, while men felt more in control of negative words. Redondo et al. (2007) preserved the original ANEW translations validated for Spain, and Sarli and Justel (2022) adjusted certain items for Argentinian usage, resulting in roughly 80% overlap, further underscoring that cultural and regional nuances shape how speakers of the same language interpret affective norms.

In addition to the direct adaptations of ANEW, several other European Spanish-language initiatives to develop affective word databases differ in methodology or scope from direct ANEW adaptations. For instance, Hinojosa et al. (2016) and Stadthagen-Gonzalez et al. (2017) focused solely on valence and arousal, finding that more negative valence correlates with higher arousal, but omitted the dominance dimension. In contrast, Ferré et al. (2017) and Pérez-Sánchez et al. (2021) employed discrete emotional categories (e.g., happiness, sadness, fear) and the concept of prototypicality. While this approach reveals category clustering, it fragments the emotional continuum into fixed classes.

In a more integrated vein also for European Spanish, Fraga et al. (2018) combined valence, arousal, and dominance with psycholinguistic properties (e.g., concreteness, familiarity) in their EmoFinder tool, though heterogeneity in data sources prevented comprehensive coverage of every dimension across all words. Meanwhile, Guasch et al. (2016) provided norms on valence, arousal, concreteness, and imageability for European Spanish, finding moderate negative correlations between emotionality and concreteness/imageability, aligning with the notion that more abstract words can carry stronger affective connotations.

In the Mexican Spanish context, Peña Pérez Negrón et al. (2018) adopted a dimensional approach; however, they deviated from the original SAM scale methodology by using emoticons to represent the dimensions and by assessing a different set of words than those typically included in the ANEW. Additionally, the instructions provided to participants focused exclusively on the positive pole of valence (e.g., “How pleasant do you find this word?”), which may bias responses and exclude neutral or negative evaluations. Additionally, the emoticons used to represent the dimensions of arousal and dominance, both of which are more complex to depict, were not clearly described, making it difficult to ascertain whether these scales accurately capture the intended constructs. This highlights the need for a fully dimensional and standardized method (valence, arousal, and dominance) for Mexican Spanish, aligned with the complete structure of the SAM.

Mexican Spanish exhibits significant regional linguistic variations, highlighting the need for culturally specific validations when working with linguistic stimuli. Words may carry unique meanings or evoke different emotional responses across Spanish-speaking countries. For instance, the term “neta,” which is widely used in Mexican Spanish to signify truth or authenticity, does not exist in Spain or Argentina. Similarly, “pasta” in Mexico refers to the Italian dish (e.g., spaghetti), but in Spain and Argentina it is also used as slang for money. Words that convey strong emotions in one region may hold neutral or alternative connotations in another. For example, “famélico” [starving], commonly understood in Spain and Argentina, is rarely used in Mexico, where “hambriento” is preferred. These linguistic and cultural differences complicate the uniform application of affective norms, even within the same language. As the largest Spanish-speaking country with over 130 million speakers (Morales, 2023), Mexico significantly influences the evolution of Spanish. The inclusion of the Mexican term “cantinflear” in the Dictionary of the Royal Spanish Academy illustrates how the language adapts to regional nuances (Real Academia Española [RAE], n.d.), emphasizing the importance of addressing these variations when designing linguistic or affective studies.

In addition to the widely documented cultural relevance in the affective assignment of valence, arousal, and dominance, it is critical to account for lexical factors such as length, frequency, and syllable count because they can systematically covary with a word’s affective properties and thus confound observed results. As shown in Larsen et al. (2008) and Estes and Adelman (2008), negative words often tend to be longer or less frequent, and without controlling for these variables, any slowdown or difference in affective ratings might be misattributed to emotional valence rather than to purely lexical factors. By measuring and controlling for word length, frequency, and syllable structure, researchers ensure that differences in affective ratings (e.g., valence or arousal) are not merely artifacts of how seldom the words appear in daily language or how cumbersome they are to process. Consequently, incorporating these objective psycholinguistic measures allows one to isolate true emotional effects from those stemming from lexical complexity or relative familiarity in the participants’ experience. Indeed, previous ANEW-based studies (Delatorre et al., 2019; Sarli & Justel, 2022) have documented the influence of such measures on affective ratings.

This study tackles these cultural and linguistic challenges by selecting words relevant to everyday language use in Mexico, ensuring that emotional ratings align with the cultural and linguistic realities of Mexican speakers. This approach provides a more reliable framework for understanding emotional expression of words in Mexican Spanish, fostering a culturally sensitive perspective on emotion in language. Thus, the aim of the present study is to provide affective ratings for 1,028 words translated from ANEW into Mexican Spanish.

## Method

### Participants

The sample comprised 753 participants (561 women, 192 men) with a mean age of 25.22 years (*SD* = 7.74). Inclusion criteria required participants to be native Mexicans, raised in the country, have Spanish as their mother tongue, and be over 18 years old. The age range of participants spanned from 18.06 to 59.90 years, with most participants in early adulthood (*n* = 708, 94%), as indicated by the interquartile range (Q1–Q3 = 20.4–26.0). Although eight participants over the age of 60 were excluded from the norms and analyses presented here, they are included in the publicly available full dataset. This allows researchers to tailor sample selection according to their specific research questions.

Additionally, participants who did not accept the informed consent were excluded, as they could not proceed with the evaluation (see Procedure). Importantly, aside from these exclusion criteria, there were no missing or invalid data. The questionnaire was designed to prevent errors: all responses were mandatory, and input formats ensured accuracy. For example, participants selected their age using a calendar, eliminating the possibility of incorrect entries.

Participants were recruited via online advertisements on social media and flyers distributed across several states in Mexico, primarily in the northwest, northeast, and central regions, which represent areas of highest population density. Participants voluntarily participated in this study and did not receive any monetary or other forms of compensation for their involvement.

### Materials

The full ANEW list (Bradley & Lang, 1999), consisting of 1,034 words, was translated into Mexican Spanish by three bilingual judges. The judges evaluated whether each word could belong to more than one grammatical category (nouns, adjectives, verbs, and foreignisms) in the context of Mexican Spanish. Additionally, they ensured that the translation into Mexican Spanish corresponded to an understandable term commonly used in Mexico.

During the first round of revision, each judge worked individually and provided their verdict. The researchers compared these verdicts and found that 75% of the words achieved full agreement among the judges. For the second round of revision, each judge was notified of the discrepancies related to the remaining 25% of the words, without disclosing specific details about the other judges'assessments. This approach allowed them to reassess the remaining words independently. After this second round, unanimous consensus was reached for the entire list.

To facilitate cross-linguistic norm comparisons, each translated word was checked against the online dictionary of the Corpus XXI of the Royal Spanish Academy (Real Academia Española (RAE), n.d.), enabling categorization into grammatical classes (nouns, adjectives, verbs, and foreignisms) and retrieval of absolute frequency and normalized frequency per million measures. Word and syllable lengths were also determined using the Syllabifier from the University Institute of Textual Analysis and Applications (Hernández-Figueroa et al., 2013).

The analysis identified 713 nouns, 243 adjectives, 65 verbs, and 13 foreignisms, some of which could function as other categories in different contexts. Additionally, some words, for example “detail,” could be interpreted as either a noun (“detalle”) or a verb (“detallar”).

Translation posed challenges for certain words due to multiple equivalents in Mexican Spanish. For instance, “table” could be translated as “mesa” or “tabla,” each with distinct contextual implications. Other words, like “muffin,” lack a direct equivalent in everyday Mexican Spanish, where the borrowed terms “cupcake” or “panqué” are commonly used. Furthermore, some words from the original list may not be widely used in Mexico, necessitating careful consideration of their relevance in this linguistic and cultural context. At the end, only 1,028 words were preserved. Approximately 86% of the words were shared between the Mexican and Spanish adaptations, while ~ 81% were shared between the Mexican and Argentinian adaptations.

### Procedure

The data were collected online using jsPsych (de Leeuw, 2015) hosted using DataPipe (de Leeuw, 2024). The study screen was designed to adapt to any display—whether a personal computer, tablet, or smartphone—ensuring that all participants had the same information arrangement.

**Fig. 1:**
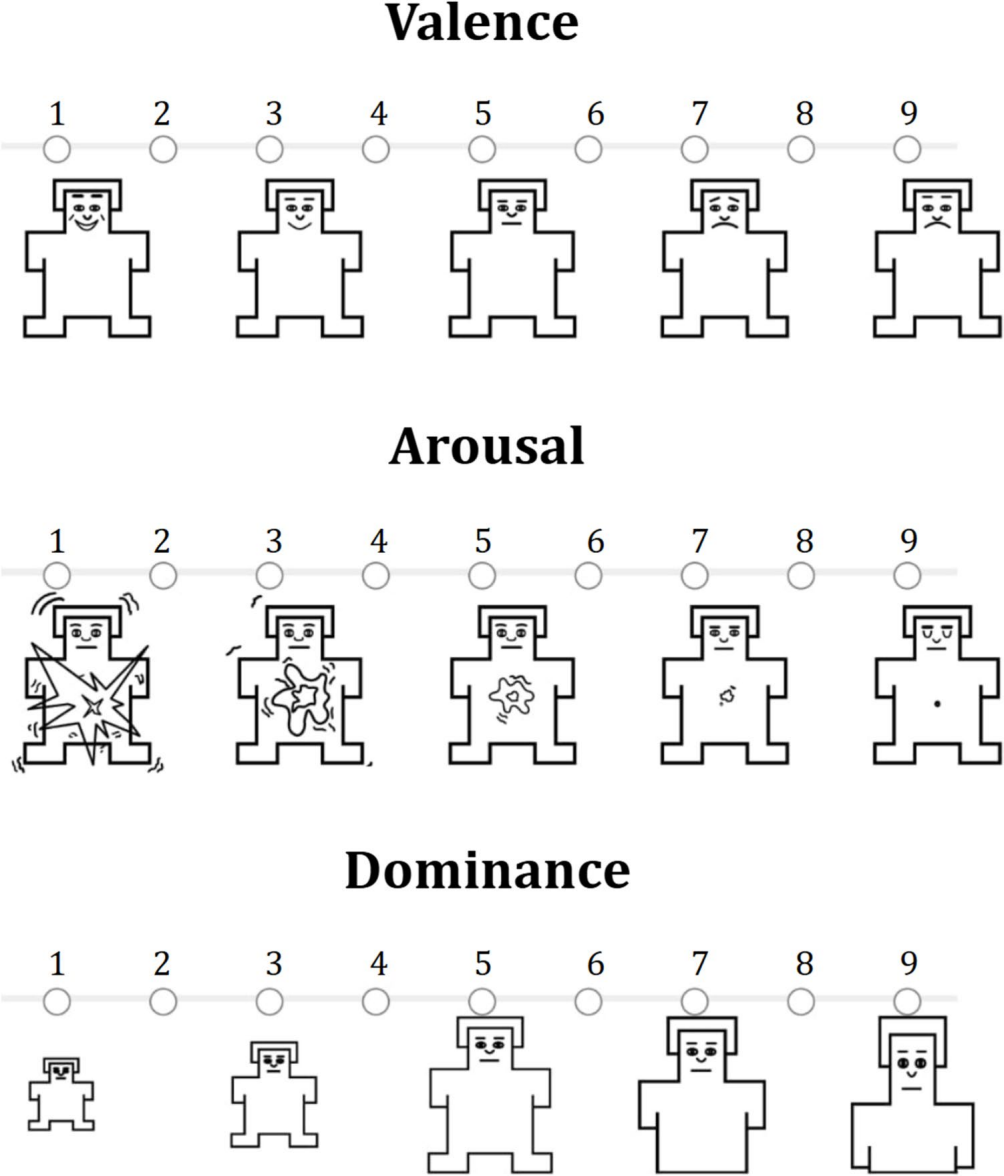
Self-Assessment Manikin (SAM). *Note:* Participants selected a position on each scale to indicate their emotional response to words across three dimensions: valence (pleasant to unpleasant), arousal (excited to calm), and dominance (out of control to in control)

Participants provided informed consent before rating each word on valence, arousal, and dominance using the nine-point SAM scale (Bradley & Lang, 1994). The instructions were primarily based on the original American version of the ANEW by Bradley and Lang (1999), with slight modifications to adapt them to the Mexican context. Instructions in Mexican Spanish were included in the Appendix. Written instructions, accompanying the visual SAM scales (Fig. 1), were presented as follows:

- General instructions: “The set of figures displayed on the screen is called SAM. You will use them to rate how you feel when reading each word. SAM represents three emotional dimensions: Pleasant vs. Unpleasant, Excited vs. Calm, and Out of Control vs. In Control. Each of these three emotions is represented on a separate scale.”
- *Valence instructions*: “This scale ranges from a smile to a frown. The leftmost figure represents feeling completely happy, content, or satisfied, while the rightmost figure represents feeling completely sad, upset, or unsatisfied. Click on the figure that best reflects how you feel. Intermediate feelings can be represented by selecting the figures between the extremes, and if you feel neutral, select the figure in the center. For more precise ratings, you can select the space between two figures.”
- *Arousal instructions*: “On this scale, if you feel highly stimulated, excited, frantic, restless, awake, or activated, select the figure on the left. If you feel very relaxed, calm, slow, apathetic, sleepy, or unactivated, select the figure on the right. For intermediate feelings of excitement or calmness, choose one of the figures in between. If you feel neither excited nor calm, select the center figure.”
- *Dominance instructions*: “Dominance reflects the level of control you feel in relation to the emotion evoked by a word. If you feel completely powerless or out of control, click the figure on the left. If you feel dominant and in control, click the figure on the right. Larger figures indicate feeling in control, while smaller ones indicate feeling controlled. If you feel neither controlled nor in control, select the center figure.”

Participants were instructed to avoid overthinking their responses, maintain a steady pace, and rely on their initial reactions. If unsure about a word’s meaning, they could either omit the rating or base it on their first impression. Before beginning the test words, participants completed three practice examples using words not included in ANEW. As shown in Fig. 1, participants rated each word on valence, arousal, and dominance on the same screen.^1^

Each participant could rate up to 96 words randomly selected from a total pool of 1,028 words. The selection ensured that no words were repeated for any participant. The 96 words were divided into four blocks of 24 words each, and the order of words within each block was also randomized. While participants could exit the task at any point, they were encouraged to complete each block in full to ensure their progress was saved.

We analyzed all questionnaires, including those from participants who responded to only one of the four presentation blocks. Since words were presented in a randomized order, partial responses do not compromise the validity of the dataset. However, data from participants who did not complete at least the first block were not saved, as responses were saved only at the end of each block.

## Results and discussion

### General description

**Fig. 2:**
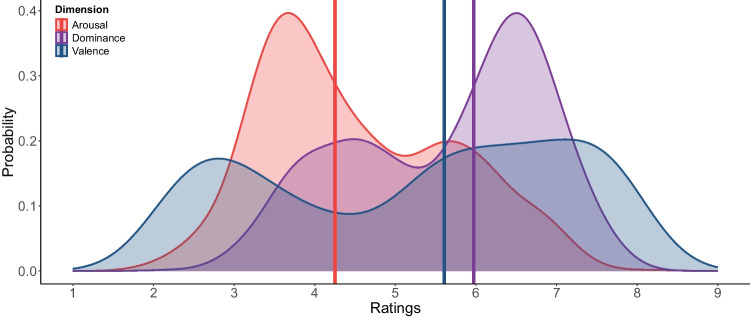
Distribution of ratings in all dimensions. *Note:* Density plots display the distributions of ratings across each affective dimension: valence, arousal, and dominance. Vertical lines mark the medians of each distribution

On average, participants evaluated 89.01 words (*SD* = 18.62, range = 24–96). However, we included participants even if they completed only one block of words, as the fully randomized word presentation ensured that this did not affect the validity of the data. Each word was evaluated by an average of 65 participants (*SD* = 7.51, range = 44–93). As shown in Fig. 2, all dimensions (valence, arousal, dominance) displayed asymmetry and a bimodal distribution. Using 5 as the neutral point, 59.72% of the words were rated with negative valence, while 40.07% were rated as positive. Similarly, only 33.75% of the words were rated as highly arousing, whereas 65.95% were rated as eliciting low arousal. Finally, 67.21% of the words were perceived as eliciting high dominance, while 32.58% were rated as having low dominance. We emphasize that using 5 is merely a convenient reference for illustrating how many words fall above or below the midpoint, rather than excluding items around 5 as “neutral.” For instance, Sarli and Justel (2022) also employed the SAM scale with nine points and found that words at or near the midpoint often reflect less extreme affective judgments, rather than a strict absence of positivity or negativity. Likewise, other recent studies (Monnier & Syssau, 2014; Soares et al., 2012) adopt ranges like 4–6 for “neutral” items, whereas Ferré et al. (2017) and Guasch et al. (2016) preserve the entire continuum without fixed cutoffs. In our case, the midpoint (5) highlights the observed asymmetry, yet all items (including those closer to 5) remain in the dataset to maintain the gradual nature of the nine-point scale.

The bimodal distribution observed across all dimensions may be linked to the nature of the responses required during word evaluation. According to Kahneman (2011), people process stimuli in either a controlled or automatic manner. Automatic processing often leads to clear, dichotomous responses (e.g., good/bad). In this study, participants classified words along a continuous spectrum with opposing poles for each dimension, while being instructed not to overthink their responses. This bimodal distribution likely reflects the quick and automatic assignment of opposing values, resulting in the observed pattern.

It is important to note that the wide age range of participants (approximately 18–60 years) may have influenced the ratings on the affective dimensions. In this regard, Fairfield et al. (2017) observed that older adults assign higher arousal and unpleasantness to certain words than younger adults, who consider those words only moderately unpleasant and less arousing. Additionally, older participants tend to perceive negative stimuli as more arousing and positive ones as less arousing, yielding a less pronounced valence–arousal curve. Older participants also display more extreme ratings for the dominance–arousal relationship, assigning lower dominance and higher arousal to words that younger adults place at intermediate levels. Therefore, the wide age variability in our sample is a limitation that should be considered when interpreting our results. However, it is important to note that 94% of the sample consisted of young adults.

### Reliability of the measures

The consistency of the collected data was measured using split-half correlations (Parsons, 2021). In this method, the full dataset was randomly divided into two subsets, and Pearson’s correlation was computed independently for each affective dimension across the two subsets. This procedure was repeated 1,000 times to calculate the average consistency. To assess the significance of these correlations, we compared the permuted correlation values against zero using a one-sample *t*-test, based on the assumption that if the data lacked consistency, the correlation would approach zero. The results revealed high correlations for all dimensions: valence (mean* r* = 0.96, *SD* = 0.001; *t*(999) = 22,997, *p* < 0.001), arousal (mean* r* = 0.89, *SD* = 0.004; *t*(999) = 6,062.2, *p* < 0.001), and dominance (mean* r* = 0.90, *SD* = 0.004; *t*(999) = 7,056.5, *p* < 0.001). These findings indicate high internal consistency.

These results demonstrate high consistency across the three dimensions. Valence exhibited clear and stable evaluations, indicating that participants easily identified words as positive or negative. Arousal, while more influenced by personal and contextual factors, remained consistent at the group level, reflecting shared perceptions of emotional intensity. Dominance, despite being a more complex dimension, also showed significant agreement, suggesting participants had a similar sense of control associated with each stimulus. These findings underscore robust stability in emotional evaluations, despite the subjective nature of each dimension, aligning with the high internal consistency reported by Redondo et al. (2007), Soares et al. (2012), and Sarli and Justel (2022). While arousal and dominance showed slightly greater variability than valence, their correlation values indicate that they remain stable dimensions. This pattern, observed in previous adaptations, suggests that valence is identified with greater clarity and direction, whereas arousal and dominance are more influenced by individual and cultural factors.

### Cross-linguistic norm comparisons

Comparing affective norms across different languages is crucial for evaluating the cross-cultural validity of emotional dimensions. These comparisons help determine whether the dimensions are universal or culture-specific. Additionally, such analyses reveal cultural differences in emotional perception and facilitate the adaptation of linguistic resources to ensure their relevance and appropriateness across various cultural contexts.

To assess this, we computed Pearson correlations across four sets of affective norms: the original norms from the USA, Portuguese norms, and two Spanish norms from Spain and Argentina. These adaptations were selected to allow for a comprehensive comparison: the Spanish norms build upon previous adaptations, the English norms serve as the original reference, and the inclusion of Portuguese provides insights into cross-linguistic similarities within a closely related Romance language.

**Fig. 3:**
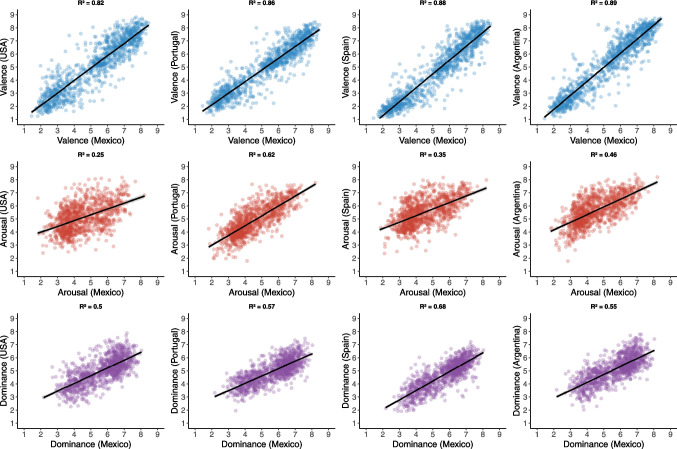
Cross-linguistic correlations

As shown in Fig. 3, the Mexican norms exhibited strong correlations with all other norms in the valence dimension (USA: *r* = 0.90; Portugal: *r* = 0.92; Spain: *r* = 0.93; Argentina: *r* = 0.94). In the arousal dimension, correlations between the Mexican norms and other languages were moderate (USA: *r* = 0.50; Portugal: *r* = 0.78; Spain: *r* = 0.59; Argentina: *r* = 0.67). Lastly, in the dominance dimension, correlations between the Mexican norms and other languages ranged from moderate to strong (USA: *r* = 0.70; Portugal: *r* = 0.75; Spain: *r* = 0.82; Argentina: *r* = 0.74).

The high consistencies observed in the valence dimension across our findings and those of the evaluated countries suggest that the ability to distinguish between pleasant and unpleasant emotions is universal across the studied cultures. This supports the notion that emotional valence is a cross-cultural dimension of emotions, emerging independently of language and enabling the identification of pleasant and unpleasant stimuli (Russell, 1991). These findings also align with research indicating that while the emotional value assigned to a stimulus is often consistent across cultures, factors such as intensity, social modulation, and context of use are significantly shaped by cultural influences (Hareli et al., 2015; Matsumoto et al., 2010).

Moderate correlations in the arousal dimension suggest that cultural context affects the perceived intensity of emotional stimuli. This may relate to cultural preferences for specific emotional states, with Western cultures often favoring high-arousal emotions compared to Eastern cultures (Tsai et al., 2006). In contrast, the dominance dimension appears to reflect the social context evoked by words, such as conflict, collaboration, or competition, as these dynamics inherently involve authority and influence (Russell, 1991).

Our findings confirm that valence exhibits robust cross-cultural stability, while arousal and dominance display greater variability. Consistent with previous adaptations of ANEW in Spanish (Redondo et al., 2007; Soares et al., 2012) and Portuguese (Kristensen et al., 2011; Soares et al., 2012), we found strong correlations in valence but more moderate correlations in arousal and dominance. For example, Redondo et al. (2007) reported that Spanish participants rated words as less pleasant yet more arousing than did Americans, while Sarli and Justel (2022) showed that Argentine participants considered negative words more arousing and felt higher dominance than their Spanish or American counterparts. Regarding Portuguese, Soares et al. (2012) found generally lower arousal ratings than in ANEW for American English, while Kristensen et al. (2011) identified specific discrepancies in emotional interpretation for Brazilian Portuguese.

Even within the same language, cultural factors emerge. European Portuguese speakers reported a lower sense of control (dominance) than did Americans (Soares et al., 2012), whereas Brazilian Portuguese participants’ ratings more closely resembled American norms (Kristensen et al., 2011). Similarly, in Spanish, Argentinian participants found negative and neutral words more arousing and felt greater control over negative stimuli, whereas European Spanish speakers perceived positive words as more arousing and reported less dominance for negative ones (Redondo et al., 2007; Sarli & Justel, 2022). Our Mexican Spanish data align with these patterns: valence correlations remain high relative to other languages and Spanish variants, yet the arousal and dominance dimensions exhibit moderate correlations, suggesting notable cultural specificity in emotional intensity and perceived control, especially for negative words. Nonetheless, the increased arousal observed for negative words in Mexico did not match the magnitude reported in Argentina, indicating nuanced regional differences.

From a methodological standpoint, comparing arousal across different sets of norms requires careful attention to how participants conceptualize emotional intensity. Indeed, various cross-cultural studies (Matsumoto et al., 2010; Russell, 1991) have shown that dimensions beyond valence, such as arousal and dominance, can generate greater variability between cultural groups, in part because language-specific factors and social norms influence how individuals interpret emotional intensity and perceived control. Nevertheless, as demonstrated in multiple studies assessing affective ratings for different types of stimuli (Bradley & Lang, 1999; Redondo et al., 2007; Warriner et al., 2013), the arousal and dominance dimensions exhibit higher interindividual differences than valence; this indicates greater variability in these ratings, which may be associated with the increased difficulty individuals face in understanding these dimensions and providing precise evaluations. Therefore, there is a need in the field to develop new methodological controls to ensure a proper understanding of these dimensions and fully address the causes of variability.

Overall, these results underscore the importance of having region-specific affective databases that measure all three core affective dimensions in a standardized fashion. While valence alone achieves high intercultural consistency, arousal and dominance can diverge substantially and may not be captured accurately without culturally adapted translations and reference norms. Our Mexican Spanish adaptation addresses these gaps by accounting for local linguistic nuances and by providing complete dimensional data, thereby enabling more precise cross-linguistic comparisons of emotional word processing.

## Bidimensional analysis

Two-dimensional analyses, particularly between valence and arousal, are usually conducted to explore the relationship between these core emotional dimensions. These analyses offer insights into how positive or negative emotions correspond to varying levels of other dimensions. This approach is important for understanding the structure of emotional experiences and identifying common patterns, such as the inverted U-shaped curve, where words with extreme valence are often associated with high arousal.

To investigate the relationship between dimensions, we first conducted both linear and quadratic regressions using a polynomial approach, including all words without splitting them by valence, arousal, or dominance. We then evaluated whether the addition of the quadratic term significantly improved model fit. This analysis aimed to capture the classic U-shaped curve typically reported in this type of validation study.

As a second step, we split the data at a reference point of 5 to distinguish between low and high values of the independent variable. We then performed linear regressions on each subset to assess the symmetry of the dimensions. This approach provides a more detailed characterization of the potential U-shaped relationship. If such a relationship exists, the slopes should differ in direction while maintaining similar strength.

## Valence and arousal

**Fig. 4:**
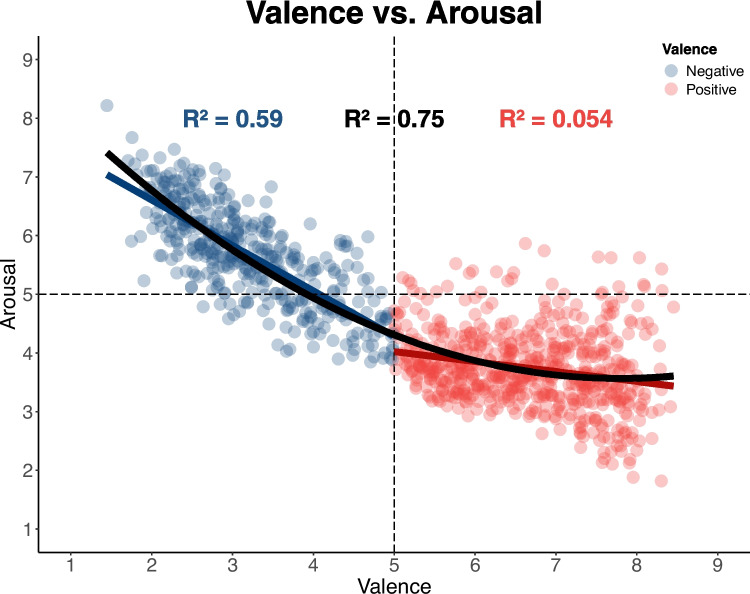
Relationship between valence and arousal. Note: The plot illustrates the relationship between valence (*x*-axis) and arousal (*y*-axis) for two stimulus groups: negative (blue points) and positive (red points). Each point represents a stimulus rated on both dimensions. The black curve shows the fitted quadratic regression line, capturing the overall trend, while blue and red linear regression lines represent trends for negative and positive stimuli, respectively. Dashed lines indicate the “neutral” point on the Likert scale

In the valence versus arousal evaluation, valence was used as the independent variable. Both the linear (β = − 0.52; *SE* = 0.01; *t* = − 48.89; *p* < 0.001; *R*^2^ = 0.69) and quadratic (β = 8.86; *SE* = 0.01; *t* = 0.59; *p* < 0.001; *R*^2^ = 0.75) models were significant; however, the inclusion of the quadratic term significantly improved the model fit (*F*(1, 78.51) = 221.85, *p* < 0.001), indicating a U-shaped relationship between valence and arousal. Despite this, the slopes for negative (β = − 0.77; *SE* = 0.03; *t* = − 24.35; *p* < 0.001; *R*^2^ = 0.59) and positive (β = − 0.16; *SE* = 0.02; *t* = − 5.89; *p* < 0.001; *R*^2^ = 0.05) valence revealed a significant negative slope, demonstrating an asymmetry in arousal values, with negative valence being associated with higher arousal compared to positive valence (Fig. 4).

## Valence and dominance

**Fig. 5:**
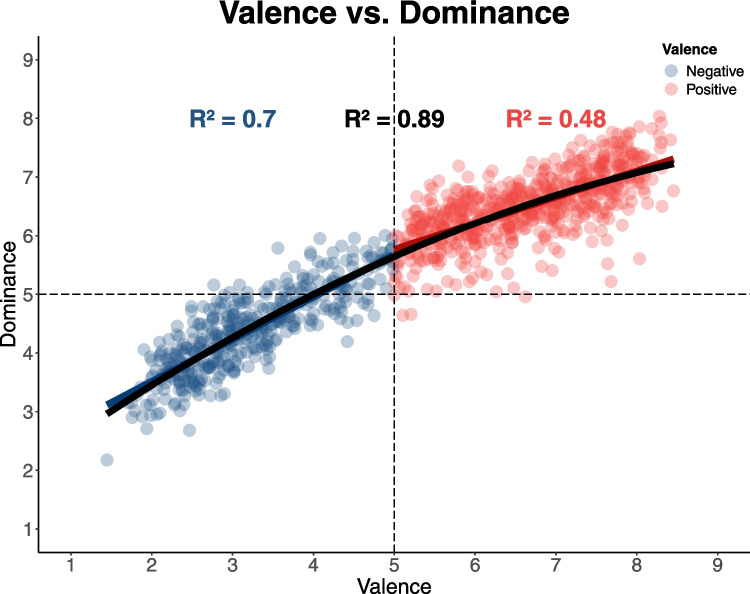
Relationship between valence and dominance. *Note:* The plot illustrates the relationship between valence (*x*-axis) and dominance (*y*-axis) for two groups of stimuli: negative (blue points) and positive (red points). Each point represents a stimulus rated on both dimensions. The black curve indicates the fitted quadratic regression line, capturing the overall trend, while blue and red linear regression lines represent trends for negative and positive stimuli, respectively. Dashed lines denote the “neutral” point on the Likert scale

In the valence versus dominance evaluation, valence was used as the independent variable. Both the linear (β = 0.60; *SE* = 0.006; *t* = 87.38; *p* < 0.001; *R*^2^ = 0.88) and quadratic (β = − 3.91; *SE* = 0.40; *t* = − 9.78; *p* < 0.001; *R*^2^ = 0.89) models were significant; however, the inclusion of the quadratic term significantly improved the model fit (*F*(1, 15.32) = 95.71, *p* < 0.001), indicating an inverted U-shaped relationship between valence and dominance. Despite this, the slopes for negative (β = 0.72; *SE* = 0.07; *t* = 27.21; *p* < 0.001; *R*^2^ = 0.70) and positive valence (β = 0.44; *SE* = 0.01; *t* = 23.89; *p* < 0.001; *R*^2^ = 0.48) were both positive, indicating an asymmetry in dominance values. Thus, positive valence was associated with higher dominance, while negative valence was linked to lower levels of dominance (Fig. 5).

## Dominance and arousal

**Fig. 6:**
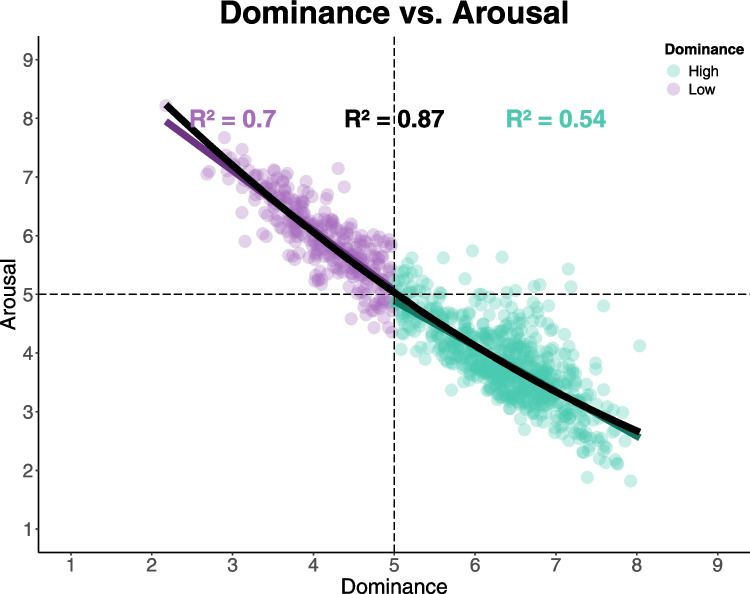
Relationship between dominance and arousal. *Note:* The plot illustrates the relationship between dominance (*x*-axis) and arousal (*y*-axis) for two groups of stimuli: low (blue points) and high (red points) dominance. Each point represents a stimulus rated on both dimensions. The black curve shows the fitted quadratic regression line, capturing the overall trend, while blue and red linear regression lines represent the trends for low and high dominance stimuli, respectively. Dashed lines indicate the “neutral” point on the Likert scale

In the dominance versus arousal evaluation, dominance was used as the independent variable. Both the linear (β = − 0.91; *SE* = 0.01; *t* = − 82.46; *p* < 0.001; *R*^2^ = 0.86) and quadratic (β = 2.65; *SE* = 0.42; *t* = − 6.23; *p* < 0.001; *R*^2^ = 0.87) models were significant; however, the inclusion of the quadratic term significantly improved the model fit (*F*(1, 7.04) = 38.88, *p* < 0.001), indicating a U-shaped relationship between dominance and arousal. Despite this, the slopes for low (β = − 1.01; *SE* = 0.03; *t* = − 27.55; *p* < 0.001; *R*^2^ = 0.69) and high dominance (β = − 0.76; *SE* = 0.02; *t* = − 28.52; *p* < 0.001; *R*^2^ = 0.54) were both negative, indicating an asymmetry in arousal values. Thus, high dominance was associated with low values of arousal, while low dominance was linked to higher levels of arousal (Fig. 6).

The finding of a U-shaped relationship between valence and arousal indicates that stimuli with extreme valence values elicit higher arousal levels, whereas words with intermediate or neutral valence evoke lower arousal. This curvilinear relationship aligns with Bradley and Lang’s (1999) observation that words with extreme valence ratings are inherently more arousing, possibly due to the heightened relevance of potentially threatening or rewarding stimuli. Furthermore, the observed emotional asymmetry, where negative words generate higher arousal than positive ones, supports the hypothesis that unpleasant stimuli provoke stronger emotional responses by activating defensive systems of the peripheral nervous system. These systems serve critical motivational functions for survival, directing responses toward protection and preservation (Bradley et al., 2001).

While the inverted U-shaped pattern in valence–arousal relationships is consistent across cultures, Mexicans rated negative words as more arousing than typically observed in other Western cultures (Redondo et al., 2007; Sarli & Justel, 2022; Soares et al., 2012). Anthropological perspectives suggest that Mexicans value open emotional expression (Garza, 1978; Guerra, 1970; Ramírez-Castaneda, 1974), potentially contributing to this heightened arousal rating. However, this cultural bias is not consistently reflected in objective measures such as behavioral or physiological responses (Naal-Ruiz et al., 2022; Soto et al., 2005), emphasizing the distinction between subjective perceptions and objective data.

The word set in this study includes a higher proportion of pleasant words. Although the valence–arousal relationship aligns with the expected boomerang pattern, pleasant words elicited lower arousal levels than the original set by Bradley and Lang (1999) and other validations (Redondo et al., 2007; Sarli & Justel, 2022; Soares et al., 2012). This trend, observed in Mexican studies of emotional stimuli from the International Affective Picture System (IAPS; Chayo-Dichy et al., 2003; Madera-Carrillo et al., 2022) and the International Dialects of English Archive (IADS; Naal-Ruiz et al., 2022), suggests that Mexicans rate unpleasant stimuli as highly arousing, while pleasant stimuli produce moderate to low arousal. Similar patterns have been observed in other cultural contexts, such as Germany, Japan, Lithuania, and Portugal (Branco et al., 2023). This highlights the importance of developing culturally specific stimuli to complement existing adaptations, ensuring homogeneity in research and clinical assessments.

The relationship between valence and dominance exhibited a clearer fit with a quadratic model, forming an inverted U-shaped curve. Dominance levels increased with higher valence values and decreased as valence declined, suggesting that pleasant emotions are associated with a greater sense of control, while unpleasant emotions correspond to diminished control. This pattern aligns with the notion that positive emotions activate appetitive motivational systems, fostering empowerment, whereas negative emotions trigger defensive systems, leading to a sense of vulnerability (Bradley et al., 2001). Although negative emotions prepare the organism to confront threats, they are accompanied by a reduced sense of control over the situation.

### Gender differences

We also conducted an analysis of gender differences to explore potential variations in emotional responses between male (*n* = 192) and female (*n* = 561) participants. Previous research has demonstrated that men and women may differ in how they experience and report emotions, particularly in dimensions such as valence, arousal, and dominance (Sarli & Justel, 2022; Soares et al., 2012). Women, for example, tend to exhibit higher emotional reactivity, especially to negative stimuli, which could result in higher arousal ratings for the same stimuli relative to men (Bradley et al., 2001; Lungu et al., 2015). Additionally, gender differences in the perception of valence and dominance are well documented, with women often rating emotionally charged words as more negative and less dominant (Montefinese et al., 2014; Warriner et al., 2013).

To account for these differences, we first computed Pearson’s correlations for valence, arousal, and dominance across groups. The results indicated strong correlations across groups (valence: *r*(1,026) = 0.94, *p* < 0.001; arousal: *r*(1,026) = 0.82, *p* < 0.001; dominance: *r*(1,026) = 0.84, *p* < 0.001), suggesting that the two groups generally provided similar ratings.

**Fig. 7:**
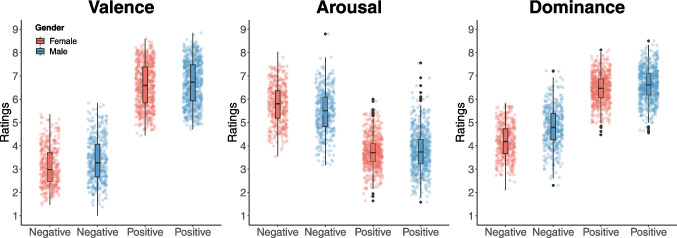
Gender differences. *Note*: The box plots display descriptive statistics for each emotional dimension, with individual data points representing each participant’s ratings. Female participants are represented in red, and male participants in blue

Additionally, we conducted a 2 × 2 mixed analysis of variance (ANOVA) to examine the interaction between gender and valence (positive or negative; Fig. 7, Table 1). Post hoc analyses focused on gender differences using independent *t*-tests. The analysis revealed a significant main effect of gender (*F*(1, 15) = 18.35, *p* < 0.001), indicating that males scored higher than females in valence. There was also a significant main effect of valence (*F*(1, 5,738) = 6,909.22, *p* < 0.001), showing that positive words received higher valence ratings than negative words. Furthermore, we found a significant interaction between gender and valence (*F*(1, 2,052) = 4.15, *p* = 0.04), suggesting that males tend to give higher ratings for both negative (*t*(1,240) = − 2.006, *p* = 0.04) and positive stimuli (*t*(799.26) = − 4.43, *p* < 0.001).

**Table 1:** Descriptive results for the gender comparison

Gender	Valence	Valence	Activation	Dominance
		Mean (*SD*)	Mean (*SD*)	Mean (*SD*)
Female	Negative	3.10 (0.822)	5.79 (0.844)	4.20 (0.728)
	Positive	6.60 (0.934)	3.73 (0.674)	6.45 (0.606)
Male	Negative	3.37 (0.933)	5.46 (0.913)	4.80 (0.848)
	Positive	6.70 (0.930)	3.79 (0.839)	6.61 (0.684)

Similarly, the analysis of the arousal ratings revealed a significant main effect of gender (*F*(1, 4.8) = 7.30, *p* = 0.006), indicating that males scored higher than females in arousal. There was also a significant main effect of valence (*F*(1, 1,715.4) = 2,614.88, *p* < 0.001), showing that negative words received higher arousal ratings than positive ones. Furthermore, a significant interaction was found between gender and valence in the arousal dimension (*F*(1, 2,052) = 27.13, *p* < 0.01), indicating that females rated negative words (*t*(806.93) = 5.29, *p* < 0.001) as more arousing than males, while no significant difference was found for positive words (*t*(1,184.9) = − 1.25, *p* = 0.21).

Lastly, for the dominance dimension, the analysis revealed a significant main effect of gender (*F*(1, 59.1) = 118.4, *p* < 0.001), indicating that females scored higher than males in dominance. There was also a significant main effect of valence (*F*(1, 2,023.1) = 4,050.48, *p* < 0.001), showing that positive words received higher dominance ratings than negative ones. Furthermore, there was a significant interaction between gender and valence (*F*(1, 2,052) = 47.59, *p* = 0.51), showing that males rated both negative (*t*(793.87) = − 210.91, *p* < 0.001) and positive words (*t*(1,222.1) = − 4.49, *p* < 0.001) as higher in dominance.

The high correlation between genders indicates that, despite specific differences, the two genders share a common perception of the basic emotional dimensions. This aligns with previous studies demonstrating significant correlations in emotional evaluations between men and women, even across cultures (Redondo et al., 2007). These findings suggest a shared structure in the perception of pleasant and unpleasant emotions across genders. The interaction between gender and valence, where men rated extreme values on both emotional poles (negative and positive) more highly, may reflect the influence of gender stereotypes and hormonal factors on emotional interpretation and expression. Such factors might lead men to externalize emotions in a more categorical and binary manner, while women may interpret emotions with greater nuance across a spectrum (Barrett, 2006; Cahill, 2006).

Additionally, women rated negative words as more arousing than men, a pattern not observed with positive words. This is consistent with evidence that women exhibit greater emotional reactivity, including more intense physiological responses, particularly to negative stimuli (Bradley et al., 2001). Women are also often socialized in environments that encourage emotional expressiveness and attentiveness to emotional cues in their surroundings (Brody & Hall, 2008). Nevertheless, a limitation of the present study is the significant disparity in the number of male and female participants, so these interpretations should be approached with caution.

### Relationships with objective linguistic measures

For each affective dimension, we performed multiple regressions that included the main effects and interactions of all psycholinguistic variables. Interestingly, only word length significantly impacted the three affective dimensions (valence: *p* = 0.04; arousal: *p* < 0.001; dominance: *p* = 0.009). Longer words were rated more negatively, as more arousing, and with lower dominance. No other predictor reached statistical significance (see Table 2 for complete statistical results of the multiple regression).

**Table 2:** Models for objective psycholinguistic measures

Factor	Valence				Arousal				Dominance			
	β	*SE*	*t*	*p*	β	*SE*	*t*	*p*	β	*SE*	*t*	*p*
**(Intercept)**	**7.532**	**1.699**	**4.433**	** < 0.001**	**2.327**	**1.067**	**2.18**	** < 0.001**	**7.907**	**1.09**	**7.255**	** < 0.001**
Syllables	0.234	0.671	0.349	0.726	− 0.102	0.421	− 0.243	0.807	− 0.131	0.43	− 0.306	0.759
**Length**	** − 0.541**	**0.274**	** − 1.976**	**0.048**	**0.538**	**0.172**	**3.126**	** < 0.001**	** − 0.458**	**0.175**	** − 2.608**	**0.009**
Frequency	0.013	0.026	0.522	0.601	0.01	0.016	0.667	0.504	− 0.001	0.016	− 0.079	0.937
Categories	− 0.277	1.184	− 0.234	0.814	0.495	0.743	0.666	0.505	− 0.608	0.759	− 0.802	0.422
Syllables:Length	0.043	0.073	0.598	0.549	− 0.067	0.046	− 1.461	0.144	0.068	0.047	1.464	0.143
Syllables:Frequency	− 0.006	0.011	− 0.564	0.572	− 0.004	0.006	− 0.591	0.554	< 0.001	0.007	0.095	0.924
Length:Frequency	− 0.001	0.004	− 0.346	0.729	− 0.001	0.002	− 0.661	0.509	< 0.001	0.002	− 0.075	0.94
Syllables:Categories	− 0.354	0.475	− 0.744	0.457	0.229	0.298	0.769	0.442	− 0.062	0.305	− 0.203	0.838
Length:Categories	0.116	0.198	0.588	0.556	− 0.189	0.124	− 1.523	0.127	0.144	0.127	1.133	0.257
Frequency:Categories	− 0.005	0.02	− 0.27	0.786	− 0.01	0.012	− 0.832	0.405	0.002	0.013	0.225	0.822
Syllables:Length:Frequency	< 0.001	0.001	0.485	0.627	< 0.001	< 0.001	0.758	0.448	< 0.001	0.001	− 0.004	0.997
Syllables:Length:Categories	0.01	0.053	0.202	0.839	0.018	0.033	0.544	0.586	− 0.02	0.034	− 0.587	0.557
Syllables:Frequency:Categories	0.004	0.008	0.487	0.626	0.003	0.005	0.682	0.495	< 0.001	0.005	− 0.103	0.918
Length:Frequency:Categories	< 0.001	0.003	− 0.115	0.908	0.002	0.002	0.999	0.317	< 0.001	0.002	− 0.34	0.733
Syllables:Length:Frequency:Categories	< 0.001	0.001	0.048	0.961	< 0.001	< 0.001	− 1.189	0.234	< 0.001	< 0.001	0.46	0.645

Bold values indicate statistical significance

Previous studies have shown that negative words are often longer and less frequent than neutral words (Larsen et al., 2008; Warriner et al., 2013). These lexical characteristics slow down word recognition (Balota et al., 2007), potentially influencing their affective ratings. Similarly, Zubicaray and Hinojosa (2024) analyzed 875 Spanish words and found that negative words tend to be longer and more frequently contain fricative and nasal phonemes, particularly in initial positions, which are linked to less pleasant or more emotionally intense sounds. In contrast, positive words were shorter and often included bilabial and velar phonemes, associated with pleasant or relaxed perceptions (Zubicaray & Hinojosa, 2024).

This relationship between word length and lower valence appears consistent across languages. Speed and Brysbaert (2024), in a study of 24,000 Dutch words, found that longer words correlated with higher arousal and emotions such as anger, fear, sadness, and surprise, while negatively correlating with joy. Additionally, positive valence in words was associated with higher frequency and prevalence (Speed & Brysbaert, 2024).

We found no significant correlation between valence and word frequency, even though Warriner et al. (2013) reported that more frequent words often exhibit more positive valence. This discrepancy may stem from a narrow frequency range in our sample, misalignment between the corpus used and participants’ actual language usage, or the overshadowing effect of a stronger predictor such as word length, which did significantly affect valence, arousal, and dominance. Future research could address this by broadening the frequency range, employing region-specific corpora, and controlling for word length more directly to confirm whether a valence–frequency relationship truly does not exist, or if methodological constraints masked it.

## Conclusion

This study highlights distinct patterns in the emotional evaluation of words within Mexican cultural norms. A higher proportion of words were rated as pleasant and low in arousal, with a tendency toward high dominance ratings. Consistent with prior findings, the emotional dimensions showed high reliability, confirming the robustness of valence, arousal, and dominance assessments. The U-shaped relationship between valence and arousal indicates that words with extreme emotional valence are perceived as more arousing, particularly negative words. Similarly, the inverted U-shaped relationship between valence and dominance suggests that pleasant words evoke a stronger sense of control, while unpleasant words are associated with a reduced sense of dominance.

Moderate correlations in arousal and dominance across languages point to cultural influences on these dimensions, emphasizing the importance of culturally relevant stimuli. Gender differences further highlight variations in arousal responses, with females rating negative words as more arousing than males, consistent with evidence of heightened female emotional reactivity. Additionally, the impact of word length on valence, arousal, and dominance underscores the role of linguistic factors in shaping emotional perception.

These findings underscore the need for culturally specific word sets tailored to Mexican contexts, particularly to identify pleasant words with higher arousal levels, which could improve the accuracy of emotional assessments within this population.

## Data Availability

The datasets generated and analyzed during the current study are publicly available at Open Science Framework: https://osf.io/c8hb2/?view_only=3466be0d101f4e1a90ef9c9ac09388d6

## Appendix

1. Original Mexican Spanish Instructions for participants:

- *Instrucciones Generales:* Al conjunto de figuras que están presentes en la pantalla se le llama SAM. Las usarás para calificar cómo te sientes al leer cada palabra. SAM muestra tres tipos de emociones: Placentero vs. Displacentero, Exaltado vs. Calmado y En descontrol vs. En control. Fíjate que cada uno de los tres sentimientos se muestra en una escala diferente.
- *Instrucciones de valencia:* Esta escala va desde una sonrisa hasta un ceño fruncido. El extremo izquierdo indica cuando te sientes completamente feliz, contento, satisfecho. El extremo derecho es para cuando te sientes completamente triste, molesto, insatisfecho. Debes dar click en la figura que mejor represente lo que sientes. Las figuras también te permiten describir sentimientos intermedios de placer, marcando cualquiera de las otras imágenes. Si te sientes completamente neutral, ni feliz ni triste, marca la figura del centro. Si tus sentimientos oscilan entre dos imágenes, marca elo entre ellas. Esto te permite hacer calificaciones más detalladas de tus sentimientos ante cada palabra.
- *Instrucciones de Activación:* En esta escala, si te sientes completamente estimulado, emocionado, frenético, inquieto, despierto o activado, da click en la figura de la izquierda. Si te sientes completamente relajado, calmado, lento, apático, soñoliento o no activado, da click en la figura de la derecha. Al igual que con la escala anterior, si presentas niveles intermedios de excitación o calma marca los espacios entre figuras. Si no te sientes ni emocionado ni calmado, marca la figura del centro.
- *Instrucciones de Dominancia:* La dominancia se refiere a la sensación de control que experimentas en relación con el sentimiento provocado por una palabra. Si te sientes completamente impotente o sin control, da click en la figura de la izquierda. Si te sientes dominante y en control, da click en la figura de la derecha. Nota que cuando la figura es grande, te sientes en control, y será muy pequeña cuando te sientas controlado. Si no te sientes ni en control ni controlado, marca la figura del centro.

## Footnotes

1 It is important to note that displaying the valence, arousal, and dominance dimensions on the same screen for each word might have influenced the ratings; however, we followed the original presentation format.

## Code availability

The code used for data analysis and figure generation in this study is publicly available at: https://osf.io/c8hb2/?view_only=3466be0d101f4e1a90ef9c9ac09388d6

## Declarations

### Author Note

The authors thank participants and caregivers for their invaluable contribution to this work.
